# Perceptual judgments for the softness of materials under indentation

**DOI:** 10.1038/s41598-022-05864-x

**Published:** 2022-02-02

**Authors:** Yusuke Ujitoko, Takahiro Kawabe

**Affiliations:** NTT Communication Science Laboratories, Nippon Telegraph and Telephone Corporation, Atsugi, 243-0198 Japan

**Keywords:** Psychology, Human behaviour

## Abstract

Humans can judge the softness of elastic materials through only visual cues. However, factors contributing to the judgment of visual softness are not yet fully understood. We conducted a psychophysical experiment to determine which factors and motion features contribute to the apparent softness of materials. Observers watched video clips in which materials were indented from the top surface to a certain depth, and reported the apparent softness of the materials. The depth and speed of indentation were systematically manipulated. As physical characteristics of materials, compliance was also controlled. It was found that higher indentation speeds resulted in larger softness rating scores and the variation with the indentation speed was successfully explained by the image motion speed. The indentation depth had a powerful effect on the softness rating scores and the variation with the indentation depth was consistently explained by motion features related to overall deformation. Higher material compliance resulted in higher softness rating scores and these variation with the material compliance can be explained also by overall deformation. We conclude that the brain makes visual judgments about the softness of materials under indentation on the basis of the motion speed and deformation magnitude.

## Introduction

A material’s softness relates to its ability to deform under pressure. The most basic measure is a material’s stiffness, which is the ratio of the force applied to the material and the amount of resulting deformation in the direction of the applied force. The same measure can also be expressed as compliance, which is the inverse of stiffness. The compliance (or stiffness) depends on the physical characteristics and geometry of the material. In a real-life situation, it should be noted that a real material’s compliance need not be a single value and it depends on various factors such as applied force. It is also known that compliance generally decreases when the strain rate, deformation of a material with respect to time, increases. It is because the applied force should be larger to overcome the increased viscous force as the strain rate increases. As strain rate increases, the material deformation is less likely to be elastic (i.e., return to its original shape) and more likely to be plastic (i.e., the deformation is permanent) or cause material embrittlement.

Softness is the subjective impression of the physical compliance of materials^1^. The mechanism for human softness judgment is complex and is not yet completely understood. How humans judge the softness of materials using haptic cues has been well investigated^2–8^. As humans do not have dedicated mechanoreceptors specialized for directly measuring a material’s compliance, a common hypothesis is that softness is judged by kinesthetic cues such as force and indentation depth, and cutaneous cues such as force distribution and contact area.

In addition to haptic cues, some visual information can serve as cues contributing to the softness judgment. A series of studies have reported that vision and haptics contribute to softness judgment in a cross-modal manner. For example, in Varadharajan et al.’s experiment^6^, there is a report that the discrimination between different compliance was better when both haptic and visual cues were present than when only one type of cues was available. They asked participants to judge the softness of virtual springs using a force feedback device with visual feedback. They found that compliance discrimination performance was improved by adding visual feedback during the compression of the virtual springs by the participants using the device. In the haptic-only condition, wherein visual feedback was excluded, the just noticeable difference increased by over 20% relative to the combined visual-haptic conditions. In contrast to that study^6^, it was found that there are individual differences and some participants did better when either only haptic or visual cue existed^9^. This could be due to differences in sensory weighting in each participant (i.e., focusing more on haptic than vision, or vice versa). It is also known that participants based their judgment, for the most part, on the visual information when there was an inconsistency between haptic and visual cues^10–12^. In Lecuyer et al.’s work^10^, the participants felt a virtual spring to be softer when the spring on the screen was compressed to a larger extent. This illusion of softness was caused in the case where participants grasped and indented a piston to apply a force to material that had specific compliance while participants were visually presented with the spring’s compression which was larger than actual. In a situation wherein participants pressed a cushion with their fingers, Punpongsanon et al.^12^ superimposed an exaggerated deformation pattern on the cushion surface by light projection. As a result, their technique successfully gave users the impression of a softer cushion. As described, it has been investigated how softness is judged when both haptic and visual cues about softness are present^13^.

Even with only visual cues, humans can discriminate between differences in the softness of materials. For example, observers can differentiate softness of materials which are dropped from above to grounds ^14,15^. Moreover, the softness of materials which are pressed by the finger of another person^13,16^ or an external object^17^ can be visually discriminated. Some previous studies have consistently reported that the indentation depth is a critical cue for observers when judging the softness of an elastic material^16,17^. In Fakhoury et al.’s experiment^16^, the participant watched video clips in which several materials having different levels of compliance were pushed by the indentor with a fixed force. The authors compared the following two conditions. In one condition, the maximum force of the indentor was fixed for all materials, and hence, the indentation depth was greater for more compliant materials than for the less compliant ones. In the other condition, the indentation depth was fixed for all materials with different levels of compliance. In this scenario, the discriminability of the material softness was greater in the former than the latter conditions. The result of this previous study suggested that the indentation depth played a major role in visually discriminating the differences in the material softness. By using video clips of computer-simulated materials, Paulun et al.^17^ obtained results consistent with Fakhoury et al.’s results^16^ in that the indentation depth was a significant cue to the judgment of material softness.

In addition to the indentation depth, the indentation speed, that is, how fast the indentation is performed, is another potential factor influencing human softness judgment. However, there is no study that has ever tested how the indentation speed could modulate the softness judgment of an elastic material. Since the variation of image motion speed across consecutive frames in the video clip is related to the perception of the mechanical properties, such as elasticity of a material that is dropped to the floor from above^18^, elasticity of cloth flapping in the wind^19^, elasticity of bending rods^20^, or the viscosity of a flowing liquid^21^, there is a possibility that the variation of indentation speed, which involves the variation of image motion speed, can affect the softness judgment of material.

Also, material compliance that is often described by means of force–displacement curves is known to affect the softness judgment. In Drewing et al’s work^13^, observers could discriminate between seven materials that had different compliance, when watching another person pressing each material several times. Observers can discriminate the differences in the compliance even when the maximum force or maximum indentation depth are fixed^16^. Further, in the previous study on haptic softness^22^, participants could discriminate between materials which were different in terms of compliance by comparing the subjective softness among the materials which were non-linearly deformed. However, it is unclear how the material compliance has an influence on the effect of indentation depth and/or the indentation speed on the judgment of material softness.

In the present study, an experiment was carried out to accomplish the following four objectives. The first objective was to clarify the effect of the indentation speed on the softness of real materials in a video clip. We shot scenes in which an elastic material was pushed by an indentor moving at an extremely slow speed (1 mm/6 seconds). Using the video clips of the scenes, we manipulated the playback speed of the clips by temporally sampling video frames. Even with this manipulation, the apparent smoothness of movements in the resultant clips was preserved because the scenes of the material indentation were shot with many video frames (the indentation of 1mm depth change was shot using 180 camera frames) because of the slow speed of physical indentation. We manipulated the indentation speed over 5 levels. namely, 2, 6, 10, 14, and 18 mm/s.

The second objective was to replicate the effect of the indentation speed on the softness rating scores. To do this, we manipulated the indentation depth of video clips in 5 levels of 6, 9, 12, 15, or 18 mm. We edited the video so that the indentor pushed the material down until it reached one of the indentation depths as described above, and immediately after that, the indentor began to go back.

The third objective was to check how the material compliance influenced the softness rating scores in the presence of other factors such as the indentation speed and the indentation depth. To accomplish this, we used three types of 3D-printed materials which had different compliance, following material designs as used in the work by Piovarči et al.^22^. By using several materials with different values of compliance, we explored how the material compliance affected the effect of the indentation depth and/or the effect of the indentation speed on the softness judgment.

The fourth objective was to see how image motion features were related to the variation of the softness rating scores which occurred when we manipulated the three factors described above.

We listed the expected outcomes of our psychophysical experiment in the following ways:

- Higher indentation speeds will lead to higher softness rating scores, which is a novel finding of the present study.
- Larger indentation depths will lead to higher softness rating scores, which is a replication of the previous results^16,17^.
- The material compliance will affect the softness rating scores, which is a replication of the previous results^13,16^. Interaction between the material compliance and the indentation depth, and/or interaction between the material compliance and the indentation speed are explored.
- The effect of the indentation speed will be well described by the variation of local motion speed between two consecutive video frames in the video clip. On the other hand, the effect of the indentation depth will be described by the overall deformation magnitude. The overall deformation magnitude is obtained by calculating the norms of motion vectors between frames before and after indentation in the video clip. The relationship between motion features and material compliance is also explored.

## Results

### Brief description of experiment procedure and stimuli

**Figure 1:**
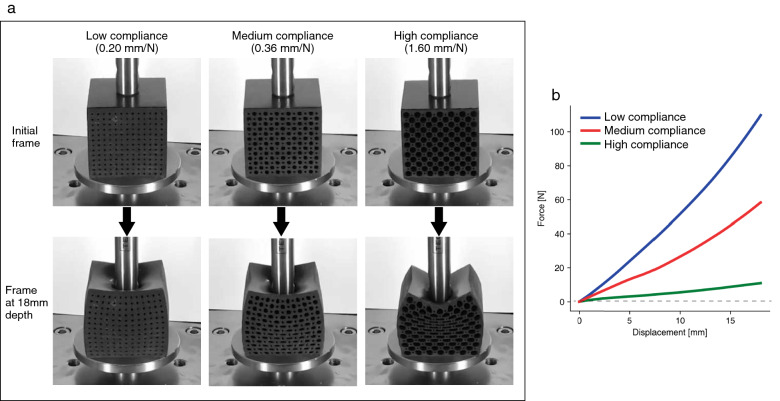
(**a**) Snapshots of video clips for materials with low, medium, and high compliance. The video frames for 0 mm and 18 mm indentation depth are shown for each material. (**b**) Force–displacement curves of three materials.

In this experiment, observers were asked to watch one of video clips in which a block of material was pressed by the indentor, and to report to what extent the material appeared to be soft on a 100-point visual analog scale. In total, there were 75 conditions which were made up of the five levels of the indentation speed $\times$ the five levels of the indentation depth $\times$ the three levels of material compliance (see the appearance of materials and force–displacement relationships in Fig. 1). We regard the material’s force–displacement relationships as linear because the R squared values of the fitted linear model that regresses the force with displacement were more than 0.988. Based on the fitted linear functions, we defined the compliance of three materials as 0.20 mm/N (low compliance material), 0.36 mm/N (medium compliance material), and 1.60 mm/N (high compliance material). Each observer reported the apparent softness for each of these conditions once.

### Softness rating scores varied with stimuli factors

**Figure 2:**
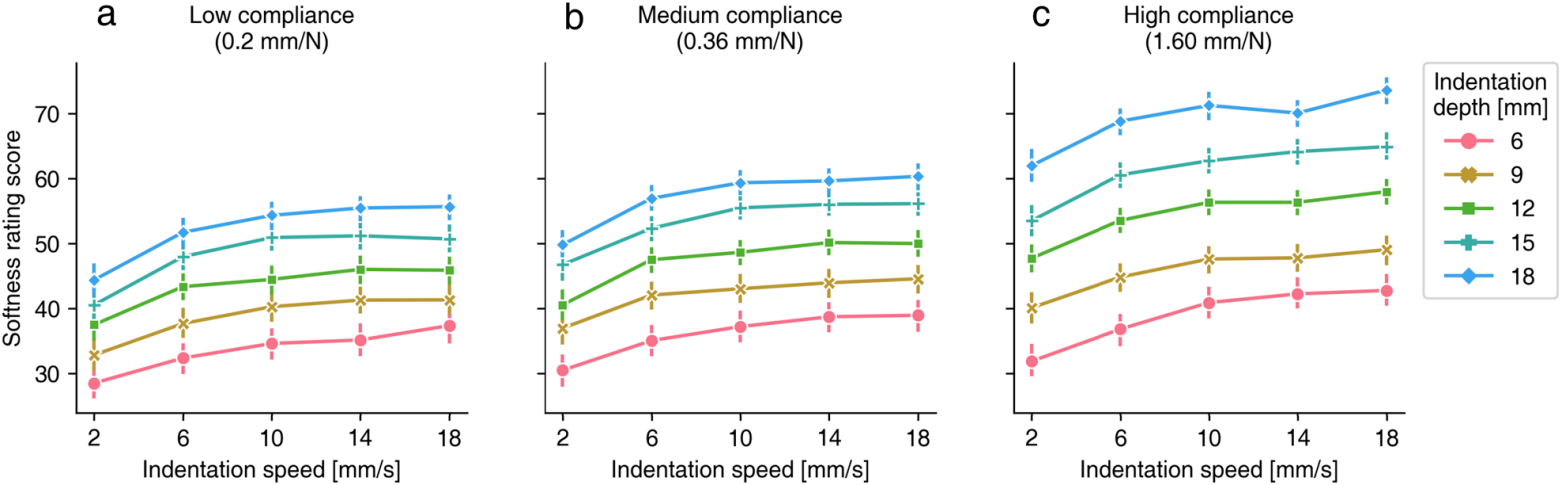
(**a**–**c**) Softness rating scores for each indentation speed and indentation depth in the case of the material with (**a**) low compliance, (**b**) medium compliance, and (**c**) high compliance. Error bars denote 95%CI.

The softness rating scores for each combination of indentation speed, indentation depth, and compliance value are shown in Fig. 2a–c.

To check whether the softness rating scores were dependent on the factors that we controlled, we performed generalized linear model (GLM) analysis. We fitted the GLM to regress the softness rating scores with the indentation depth, the indentation speed, and the material compliance as factors. Since the softness rating scores are positive continuous values that are expected not to have a normal distribution, the GLM employed a logarithmic link function with a gamma distribution.

As the result of a likelihood ratio test (Type II test), there were significant main effects of the indentation depth [$df=4, {\chi }^2=4322.7$, $p<0.001$], the indentation speed [$df=4, {\chi }^2=844.6$, $p<0.001$], and the material compliance [$df=2, {\chi }^2=1089.8$, $p<0.001$]. The interaction effect between the indentation depth and material compliance was also significant [$df=8, {\chi }^2=68.1$, $p<0.001$]. There was no significant interaction effect between the indentation speed and the material compliance [$df=8, {\chi }^2=4.7$, $p=0.79$], and no significant interaction effect between the indentation speed and the indentation depth [$df=16, {\chi }^2=17.8$, $p=0.36$].

As post-hoc tests of the significant main effects, we conducted multiple comparisons for each of the significant main effects. There were significant differences between all pairs of the indentation depth ($p<0.001$) and all pairs of the material compliance ($p<0.001$). For the pairs of the indentation speed, all pairs had significant differences ($p<0.001$), except the pairs of [10 mm/s - 14 mm/s]($p=0.28$) and [14 mm/s - 18 mm/s]($p=0.44$).

Since there was a significant interaction effect between the indentation depth and the material compliance, we conducted multiple comparisons focusing on the simple main effect of depth within each material. The result shows that every pair of the indentation depth within each level of the material compliance condition was significantly different ($p<0.001$). Also, we conducted multiple comparisons for material compliance within each level of the indentation depth. The result shows that every pair of material compliance within each level of the indentation depth conditions was significantly different ($p<0.001$).

In addition, to determine which main effect of the three factors was most significant for softness rating scores, standardized partial regression coefficients were compared. We fitted the GLM to regress the softness rating scores with the standardized indentation depth, the indentation speed, and the material compliance as continuous values. All factors were significant ($p<0.001$), and the standardized partial regression coefficient of the indentation speed with standard error was $0.068 \pm 0.0052$, that of the indentation depth was $0.186 \pm 0.0052$, and that of the material compliance was $0.083 \pm 0.0052$. This suggests that the indentation depth was more critical than the other two factors.

### Image motion features

As described, we checked the effect of the three factors, that is, the indentation depth, the indentation speed, and the material compliance, on the softness rating scores. We hypothesized that human observers rated softness using image features that changed in accordance with these factors of stimuli. The previous studies^17,18,23,24^ showed that the successive deformation of elastic materials produced variations in image features such as motion and shape, and that human observers took advantage of these features to visually judge the properties of the elastic materials. In this respect, it was necessary to check what kind of change in image features was produced by the factors tested in the experiment, and how the image features contributed to the softness judgment.

Here, we focused on image motion features. Specifically, to obtain motion vectors in the clip, we tracked the salient points of materials in video clips using Lucas–Kanade algorithm^25^. In order to eliminate the influence of noise in the background part of the image, only 14 representative points on the material area was used. These 14 points include six points in the corners and six points on the edges and one point at the center on the front surface and one point at the point of indentation (see Supplementary Fig. 1). Based on the tracking of these 14 points, we calculated two indices of motion features: local motion speed and overall deformation magnitude.

**Figure 3:**
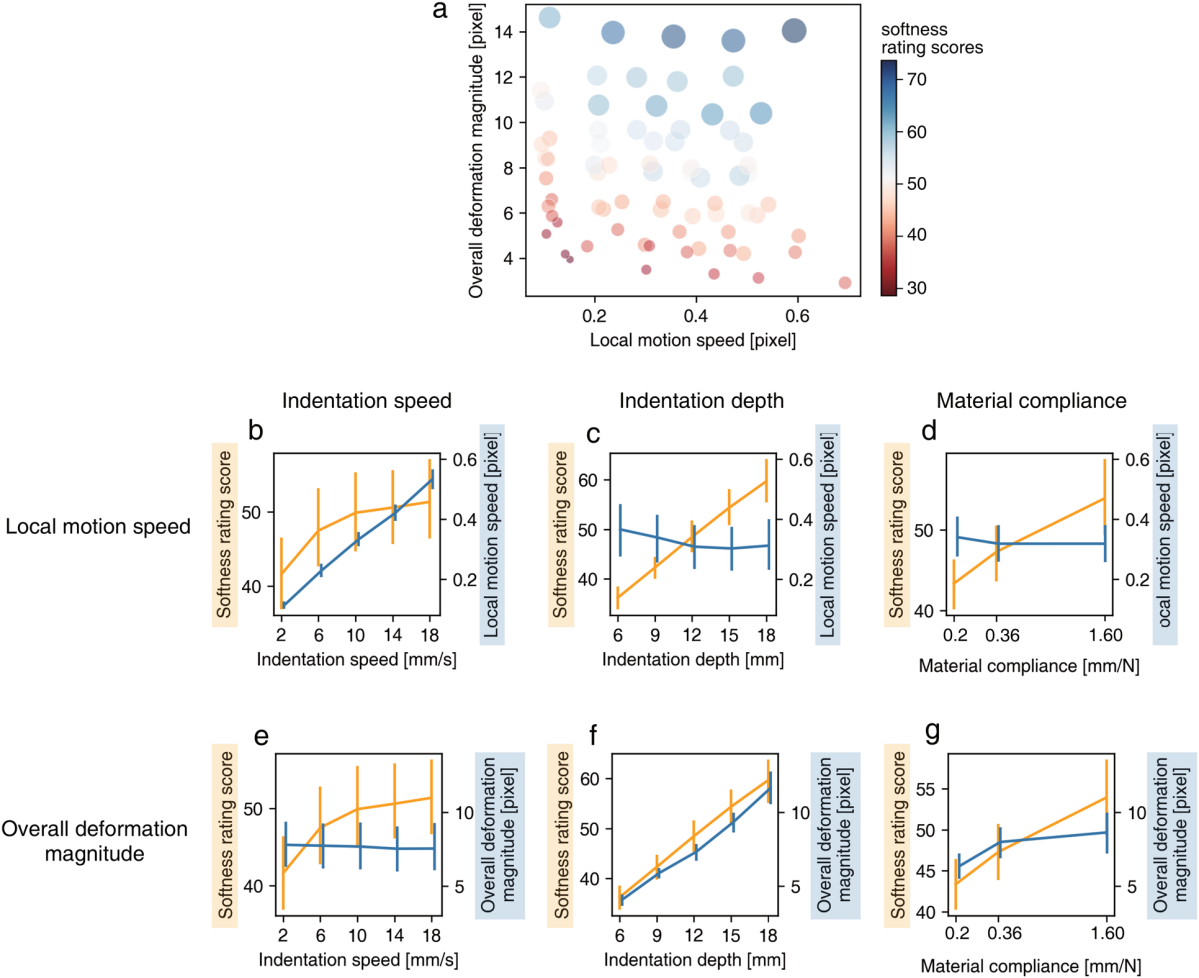
(**a**) Softness rating scores varied with image motion features. (**b**–**d**) Local motion speed as a function of (**b**) the indentation speed, (**c**) the indentation depth, and (**d**) material compliance. Blue lines denote local motion speed and orange lines denote softness rating scores. (**e**–**g**) Overall deformation magnitudes as a function of (**e**) the indentation speed, (**f**) the indentation depth, and (**g**) material compliance. Blue lines denote overall deformation magnitude and orange lines denote softness rating scores. Please note that the x-axes of (**d**) and (**g**) are a logarithmic scale. Error bars denote 95%CI.

For the local motion features, we wanted to know whether local motion speed, which has been often reported as a perceptual cue to material properties^18,21^, influenced the softness rating scores. To obtain the local motion speed, we computed the norm of the motion vector at 14 points between all two consecutive frames in video clips and averaged the norm of the vector through a single video clip. The resultant value was taken as local motion speed, as shown in Fig. 3b–d.

For the overall deformation magnitude, we wanted to know whether overall deformation magnitude, which has been also reported as a perceptual cue to material properties^17,26^, influenced the softness rating scores. To obtain the overall deformation magnitude, we calculated the norm of vectors which was calculated for the first and the middle of all frames with a maximum indentation depth, and the resultant value was taken as the overall deformation magnitude, as shown in Fig. 3e–g. Although the overall deformation magnitude was calculated on the basis of image motion, we considered it as the index of change in shape across time because the overall deformation magnitude was made by calculating norms of motion vectors between the first and the middle of all frames which were in long temporal ranges to which human motion system does not have sensitivities; it is known that human visual systems detect motion by spatiotemporally integrating luminance signals within the receptive field, which temporally spans across 100–300 msec^27,28^. Rather, the index is likely related to perceptual processing that can detect changes in shape in a long temporal range^29^.

Figure 3a shows the relationship between motion features and softness rating scores. To investigate how well the local motion speed and the overall deformation magnitude could explain the softness rating scores averaged across observers, we fitted the GLM to regress the softness rating scores with these two motion features. Nagelkerke’s pseudo R squared value was 0.883, indicating that the two motion features could explain the softness rating well. Spearman’s rank correlation between the two motion features was − 0.181 ($p=0.12$), which demonstrated that there was no significant correlation between the local motion speed and the overall deformation magnitude .

#### Relationship between local motion speed and softness rating scores

Figure 3b–d shows the relationships among the factors we tested in the experiment, the softness rating scores, and the motion features. We calculated correlation coefficients between the factors and motion features, and the softness rating scores and the motion features. Since some of the motion features did not follow a normal distribution as determined by the Shapiro-Wilk normality test, we calculated the Spearman’s correlation coefficient.

We calculated correlation coefficients between local motion speed of all video clips and the each of three factor’s levels. The correlation analyses showed that local motion speed was positively correlated with indentation speed, but was not positively correlated with indentation depth or material compliance as follows. Namely, the correlation coefficient between the local motion speed and the indentation speed was 0.97 ($p<0.001$, see a blue line in Fig. 3b). The correlation coefficient between the local motion speed and the indentation depth was − 0.13 ($p=0.261$, see a blue line in Fig. 3c). The correlation coefficient between the local motion speed and the material compliance was − 0.05 ($p=0.630$, see a blue line in Fig. 3d).

Also, we calculated correlation coefficients between local motion speed and softness rating scores aggregated in terms of each of three factors. Per each observer, we averaged the softness rating scores in terms of each of three factors’ levels, and then, the correlation coefficients were computed. The result showed that the local motion speed explained the softness rating aggregated in terms of the indentation speed, but not those aggregated in the case of indentation depth and material compliance as follows. Namely, the correlation coefficient between the local motion speed and the softness rating scores that were aggregated in terms of the indentation speed was 0.99 ($p<0.001$).The correlation coefficient between the local motion speed and the softness rating scores that were aggregated in terms of the indentation depth was -0.70 ($p=0.188$).The correlation coefficient between the local motion speed and the softness rating scores that were aggregated in terms of the material compliance was -0.50 ($p=0.667$).

#### Relationship between overall deformation magnitude and softness rating scores

We calculated correlation coefficients between overall deformation magnitude of all video clips and factor’s levels of the clips. The correlation analyses showed that deformation magnitude was positively correlated with indentation depth, but was not positively correlated with indentation speed or material compliance as follows. Namely, the correlation coefficient between the overall deformation magnitude and the indentation speed was -0.05 ($p=0.686$, see a blue line in Fig. 3e). The correlation coefficient between the overall deformation magnitude and the indentation depth was 0.93 ($p<0.001$, see a blue line in Fig. 3f). The correlation coefficient between the overall deformation magnitude and the material compliance was 0.28 ($p=0.015$, see a blue line in Fig. 3g).

Also, we calculated correlation coefficients between overall deformation magnitude and softness rating scores in terms of each of three factors. Per observer, we averaged the softness rating scores aggregated in terms of each of three factors’ levels, and then, the correlation coefficients were computed. The result showed that the overall deformation magnitude explained the softness rating aggregated in terms of the indentation depth, but not those aggregated in terms of the indentation speed and material compliance as follows. Namely, the correlation coefficient between the overall deformation magnitude and the softness rating scores that were aggregated in terms of the indentation speed was -0.9 ($p<0.001$).The correlation coefficient between the overall deformation magnitude and the softness rating scores that were aggregated in terms of the indentation depth was 1.0 ($p<0.001$). The correlation coefficient between the overall deformation magnitude and the softness rating scores that were aggregated in terms of the material compliance was 1.0 ($p<0.001$).

#### Other motion features

As compared to the present study which used motion features in the sparse optical flow, Kawabe et al.^21^ and Bi et al.^24^ used the more complex, idiosyncratic motion features based on dense motion vectors (mean and standard deviation of absolute divergence, mean and standard deviation of gradient, mean and standard deviation of discrete Laplacian). To compare our results with their results, we investigated how the idiosyncratic motion features were related to our aforementioned motion features. Supplementary Table 2 shows Spearman’s rank correlation coefficient between them. The correlation between all idiosyncratic motion features and local motion speed was high, while the correlation with overall deformation magnitude was low. Thus, it seems that the part of the results of the present study that are explained by local motion speed can be also explained by idiosyncratic motion features. Importantly, however, it should be noted that our local motion speed is based on 14 representative points of optical flow. Studies have shown that humans can extract material and scene information from motion information presented in limited positions^21,30,31^. Consistent with those studies, the results shown in the present study indicate that the brain judges the softness of the material from the motion features extracted sparsely in space. It is also known that the spatial resolution of motion extraction in the human visual system is not very high^32,33^. From these points of view, we think it is more likely that the motion features captured sparsely can describe information that is more useful to humans than the motion features captured densely. Thus, we discuss the results based on the local motion speed and overall deformation magnitude.

## Discussion

The purpose of the present study was to examine which factors and motion features determined the observer’s judgment of softness. We discovered that larger indentation speeds resulted in larger softness rating scores. Moreover, the variation of the rating scores due to the variation of the indentation speed was successfully explained by the local motion speed (see Fig. 3b). The results added new evidence to the literature of the softness perception, showing that observers can visually estimate the mechanical properties based on the local motion speed, which is tightly related to the indentation speed. Our results support the previous idea^34–36^ that the brain does not try to faithfully reconstruct the physical properties of the material but heuristically generates the representation of materials on the basis of image cues.

We made the following speculation as to why the observers heuristically estimated the material softer when the indentation speed was large. A previous study with a paradigm similar to ours showed that observers judged material softness more accurately on the basis of different indentation depths with constant force than on the basis of different forces with constant indentation depth^16^. The previous results open a possibility that observers assume that force is constant. In the current study, if the force is considered to be constant, an interpretation would be that indentations are faster in some video clips because they are “easier” to indent (i.e., the material is more compliant). In other words, the larger indentation speed should be attributed not to properties of the indentor (force) or the video (playback speed) but to properties of the material compliance. Let us consider another possibility that the observers assume different forces depending on video speed. In other words, in the possibility, observers assume that the force is stronger when the indentation speed is larger. Here we should note that assuming the speed-dependent force variation accompanies the second observer’s assumption that materials have the same compliance, which clearly contradicts our result that the judged softness varied depending on videos (Fig. 2). Thus, we believe that the assumption of constant applied force might be reasonable to explain the effect of indentation speed on softness judgments.

On the other hand, it should be noted that the assumption of constant applied force is not explicitly tested. In our experiment, we did not instruct or ask about applied force in video clips or video playback speed. Hence, it was unclear how our observers perceived force and playback speed in our stimuli. Explicitly asking force or video speed may change the softness rating scores. That is, there is a possibility that softness rating scores may change if observers are asked to report force and/or playback speed and eventually report different levels of them with different indentation speeds. Future studies need to address this possibility by asking observers to judge applied force and playback speed in addition to material softness.

It is an interesting question whether the manipulation of indentation speed could influence the realism of video clips as used in the experiment. In the previous study^37^, it has been shown that observers were not so sensitive to playback speed in natural scenes movies. As the authors of the previous study mentioned, this may be because observers attributed the speed change to material properties. Consistent with the previous study, as far as we checked the stimuli in our experiment, the realism of all video clips in our stimulus set was high. On the other hand, it would be unclear what would happen if the speed of video clips was high or low enough to contradict the observer’s expectation based on the physical law. Indeed, it is possible to deteriorate the realism of the appearance of physical phenomena. For example, it has been shown that the realism of the Poisson effect could be hampered when the physical relationship between deformations along the longitudinal and transverse axes of strain was greatly violated from^38^. To answer the question, it is necessary to ask observers to report the realism of clips as well as material softness at the same time.

Further, it should be also noted that in the stimuli of the present study, the start position of indentor’s movement was always at the top surface of the material and thus, there was no cue indicating the indentation speed was controlled to be constant. In contrast, if the start position was above the top surface, the speed change at the contact to the top surface could be the cue for whether the indentation speed was controlled or not. For example, if indentor’s speed does not change before and after surface indentation, the indentation speed will be presumably attributed to the controlled indentor rather than the material compliance. If the start position is above the top surface and the indentor’s speed change at the contact of surface, the change in the indentation speed will be presumably attributed to the material compliance. Future studies need to test the possible role of indentor’s speed changes before and after the contact to the top surface.

In addition, the effect of the indentation speed saturated at the large indentation speed while local motion speed did not. This saturation may have occurred due to the perceptual indistinguishability of the absolute difference in local motion speeds over the greater range of the indentation speed^39–41^.

Consistent with the previous studies^16,17^, we observed that the indentation depth had a strong effect on the softness judgment. Moreover, the variation of the rating scores with the indentation depth was successfully explained by the overall deformation magnitude (see Fig. 3f). Our results are in accordance with their results^16^ showing that the indentation depth rather than the applied force was critical to the discrimination of the softness of elastic materials. Although the authors of the work^16^ used several real materials that had diverse compliance characteristics, our results showed that the variation of the indentation depth caused the variation of the softness rating scores even for the samples with an identical material compliance (see Fig. 2). Our results are also in accordance with the previous results^17^ showing that the indentation depth was a strong determinant of judged stiffness, while that previous study did not test how the indentation depth had an interplay with the indentation speed and material compliance.

We also found that overall deformation magnitude could explain the variation of the material softness judgment depending on the material compliance. When the material with large compliance is indented, the deformation of the material is large and resultantly, the overall deformation magnitude becomes large. The results indicate that observers might make an estimate of softness using this overall deformation magnitude as a measure of degree of deformation that was influenced by the material compliance.

Although we showed that all three factors we tested influenced on the softness judgment, we also found that the contribution of the factors was not identical to each other. Comparing the absolute value of the standardized partial regression coefficients of factors in the GLM analysis, we found that the indentation depth had the largest coefficients among the three factors, and was followed by the material compliance and the indentation speed in order. As far as we investigated, the brain possibly uses the indentation depth as the most robust cue to the softness judgment and also uses other cues such as the material compliance and the indentation speed as relatively minor cues. With such multiple cues available, the brain likely makes judgments about material properties in a reliable manner. This means that even when a certain cue is not available for various reasons, the brain may be able to judge material properties without large errors on the basis of other available cues. This idea suggests that graceful degradation, which is one of principles of biological vision proposed by Marr^42^, may also be valid in judging material properties.

Although the local motion speed and overall deformation magnitude could successfully describe parts of softness judgments, it should be noted that these two motion features are not a general descriptor of softness judgment, which can be applicable to any type of natural scenes; it can vary with multiple factors such as the translation speed of rigid materials and the flow speed of liquids. Thus, the variation of these two motion features do not always involve the difference in softness judgment in all natural scenes. On the other hand, these two motion features are still effective to explain how the observers differentiated softness for a given set of materials. Moreover, it is also useful to describe the softness in a situation wherein a material on a plate is indented from top. Since the purpose of the present study was to specify motion features that could explain observers’ softness judgments in the situation wherein a material was indented from top, the usage of motion cues for the explanation is reasonable. On the other hand, more elaborations on motion statistics are critically necessary to clarify what motion features are used to judge softness judgements in stimulus sets wherein various types of material motion/deformation are included.

A limitation of the present study stems from the fact that we used materials that had different-sized holes to give different levels of compliance to the materials, and these holes were shown on the surface. This gave the materials different surface appearances in addition to the intended different deformations of overall structure. Our experimental design did not distinguish between the contribution of motion features from the variation of surface holes and the contribution of motion features from other sources such as the deformation of overall structure of the materials. In future studies, it may be necessary to use stimuli that eliminate the contribution of the appearance of the surface holes to the softness judgment by, for example, inpainting the holes in the video clips.

## Method

### Observers

In total, 300 people participated in the experiment. Each age group (20s, 30s, and 40s) consisted of 50 men and 50 women and the mean age was 35.3 (SD: 8.61). The participants were recruited online by a crowdsourcing research agent in Japan and were paid for their participation. Only people who could participate in the experiment using their own personal computers were recruited and they were unaware of the specific purpose of the experiment. Ethical approval for this study was obtained from the ethics committee at Nippon Telegraph and Telephone Corporation (Approval number: R02-009 by NTT Communication Science Laboratories Ethics Committee). The experiments were conducted according to the principles that have their origin in the Helsinki Declaration. Written informed consent was obtained from all observers in this study.

### Stimuli

The stimuli were video clips that showed the scenes of an elastic material pushed from the top surface by an indentor. The video resolution was 288 × 288 pixels at 29.97 frames per second.

#### 3D printed material

We attempted to replicate three cubes of metamaterials introduced in^22^ by using the identical types of material (TangoBlackPlus) and a 3D printer (Stratasys Obje500). Figure 1a,b show snapshots of the video clips for the materials A, B, and C, and their force–displacement curves. The length of each edge of the material was 42 mm. Each material contained 169 cylindrical holes. To control the material compliance, the material was structured with different-sized holes. Specifically, there were two sizes of hole for each material, and the sizes were different depending on the material as shown in Supplementary Table 1. The distance between the holes was 3.0 mm. These configurations were identical to those used in the previous study^22^. Because the cube’s surfaces in the previous study had a striped pattern and thus were not smooth, we made the surfaces flat because we wanted to remove the effect of a striped pattern on the perception of softness.

To characterize the material property, we performed uniaxial load testing. An increasing force was applied to the materials from the top to give an indentation at a constant speed of 1 mm / 6 seconds, and the corresponding force was recorded using a force tester (MCT-2150, A&D Co., Ltd.). Figure 1b shows the measured force–displacement curves. Although there was a difference in the force–displacement curve from the previous study^22^, this could be attributed to the aforementioned absence of surface striped patterns.

To see how linear the deformation of the three materials was, we fitted the data with linear models to regress the force with displacement for each material. The R squared values of the fitness were 0.993 (low compliance material), 0.988 (medium compliance material), and 0.992 (high compliance material). Thus, we regarded the force–displacement relationships as linear and defined the compliance of the three materials as 0.20 mm/N (low compliance material), 0.36 mm/N (medium compliance material), and 1.60 mm/N (high compliance material).

#### Video clips

We took videos from diagonally above so that the top surface pushed by the indentor could be clearly seen. The horizontal distance from the camera to the material was 40 cm and the height of the camera was 17 cm above the material. The elevation angle of the camera was approximately 23 $^\circ$. The camera lens was oriented towards the material. The indentor was a cylinder with a diameter of 1.3 cm.

The raw video recorded the indentor making an indentation into the material at a constant speed of 1 mm/6 seconds. The raw video started from the time when the indentor was stationary and in contact with the top surface and ended when the indentation depth reached 18 mm.

We edited the raw video so that the indentor pushed the material down to a certain depth, and immediately after that, the indentor began to go back. We prepared videos, each of which was different in terms of maximum indentation depth and indentation speed. There were five maximum indentation depth levels: 6, 9, 12, 15, 18 mm. The videos were trimmed so as not to exceed the maximum indentation depth for each depth level. The indentation speed across video frames was constant in each clip. We increased the playback speed of the original video and set five speed levels: 2, 6, 10, 14, and 18 mm/s.

In total, there were 75 conditions (3 material compliance levels $\times$ 5 depth levels $\times$ 5 speed levels).

### Procedure

The experiment was programmed using jsPsych^43^. Observers participated in the experiment in their own environments with personal computers. Neither observation distances nor screen sizes were controlled. Although the presentation accuracy was not measured, it has been reported that stimulus timing control with jsPsych is sufficient to conduct online psychological experiments^44^.

After viewing the sequence, the softness was assessed using a visual analog scale (VAS) ranging from 0 to 100 with 100 scale divisions. The “Not soft at all” anchor was placed on the left side of the scale and the “Softer than anything you can imagine.” anchor was placed on the right side of the scale. The observers were instructed to click on the point representing the perceived softness of the material shown in the video clip.

The experiment was composed of familiarization and test phases. In the familiarization phase, each observer provided answers for six trials, which were randomly extracted from the 75 conditions. After these were completed, the test phase started. In the test phase, each observer provided an answered for each of the 75 conditions. The presentation order of the 75 conditions was pseudo-randomly assigned to each observer.

### Data analysis

In order to investigate whether the softness rating scores for the 75 videos differed depending on the indentation depth, the indentation speed, and the material compliance, we conducted a GLM analysis. Since the softness rating is a positive continuous value, it was modeled by a logarithmic link function with a gamma distribution. To determine which factor was significant, a likelihood ratio test (Type II test) was performed. If there was a significant factor, we further performed multiple comparisons with a corrected alpha level ($p<0.05$) using the Bonferroni method for the significant main effect or the significant simple main effect. In addition, to determine which factor was more significant, standardized partial regression coefficients were compared by fitting the GLM to regress with standardized indentation depth, the indentation speed, and the material compliance as continuous values.

As software, we used “glm” and “emmeans” function in “R” package for GLM analysis and multiple comparisons.

### Image analysis

We computed the optical flow at 14 representative points of all consecutive frames in video clips using Lucas–Kanade algorithm^25^. In order to eliminate the influence of noise in the background part of the image, the optical flow of only 14 representative points on the material area was calculated. These 14 points include six points in the corners and six points on the edges and one point at the center on the front surface and one point at the point of indentation (see Supplementary Fig. 1). In a single video clip, the indentor pushed the material down to a certain depth, and immediately after that, the indentor began to go back. As such, the image motion speed was identical between the first half and second half of the video clip though the image motion direction was reversed between them. To reduce the redundancy, we analyzed the optical flow field by focusing on the first half. Based on the motion vectors of the optical flow fields, we calculated the following two indices of motion features: local motion speed and overall deformation magnitude. To obtain the local motion speed, we averaged the norm of the vector through a single video clip, and the resultant value was taken as the local motion speed. To obtain the overall deformation magnitude, we averaged the norm of vectors which was calculated for the first and the middle of all frames, and the resultant value was taken as the overall deformation magnitude, as shown in Fig. 3.

To determine how these motion features varied with the factors such as indentation depth, the indentation speed, and the material compliance, we calculated correlation coefficients between these motion features of all video clips and each of three factors of the clips. In addition, to determine whether observers utilize these motion features to judge the material softness, we calculated the correlation coefficient between each of the two motion features and softness rating scores aggregated in terms of each of three factors. Specifically, we aggregated the softness rating scores per observer in terms of each of three factors, and then, the correlation coefficients were computed. The significant correlation suggested that the softness judgment was made based on motion features that varied depending on the factors we manipulated in the experiment.

## Supplementary Information

Supplementary Information 1.

Supplementary Information 2.

## Data Availability

The authors confirm that the data supporting the findings of this study are available within the article and its supplementary materials.

## Code availability

The code used for the analysis in this study is available from the corresponding authors upon request.

## References

[1] Luca, M. D. *Multisensory Softness, Perceived Compliance from Multiple Sources of Information*. Springer (2014).

[2] Kuschel M, Di Luca M, Buss M, Klatzky RL (2010). Combination and integration in the perception of visual-haptic compliance information. IEEE Trans. Haptics.

[3] Pressman A, Welty LJ, Karniel A, Mussa-Ivaldi FA (2007). The international journal of robotics. Int. J. Robot. Res..

[4] Srinivasan MA, LaMotte RH (1995). Tactual discrimination of softness. J. Neurophysiol..

[5] Tiest WMB, Kappers AM (2009). Cues for haptic perception of compliance. IEEE Trans. Haptics.

[6] Varadharajan, V., Klatzky, R., Unger, B., Swendsen, R. & Hollis, R. Haptic rendering and psychophysical evaluation of a virtual three-dimensional helical spring. In *2008 Symposium on Haptic Interfaces for Virtual Environment and Teleoperator Systems* 57–64 (IEEE, 2008).

[7] LaMotte RH (2000). Softness discrimination with a tool. J. Neurophysiol..

[8] Bicchi A, Scilingo EP, De Rossi D (2000). Haptic discrimination of softness in teleoperation: The role of the contact area spread rate. IEEE Trans. Robot. Autom..

[9] Gurari, N., Kuchenbecker, K. J. & Okamura, A. M. Stiffness discrimination with visual and proprioceptive cues. In *World Haptics 2009-Third Joint EuroHaptics Conference and Symposium on Haptic Interfaces for Virtual Environment and Teleoperator Systems* 121–126 (IEEE, 2009).

[10] Lécuyer, A., Coquillart, S., Kheddar, A., Richard, P. & Coiffet, P. Pseudo-haptic feedback: Can isometric input devices simulate force feedback? In *Proceedings IEEE Virtual Reality 2000 (Cat. No. 00CB37048)* 83–90 (IEEE, 2000).

[11] Tatezono, M. *et al.* Effect of haptic feedback on pseudo-haptic feedback for arm display. In *2009 ICCAS-SICE* 4332–4337 (IEEE, 2009).

[12] Punpongsanon P, Iwai D, Sato K (2015). Softar: Visually manipulating haptic softness perception in spatial augmented reality. IEEE Trans. Vis. Comput. Graph..

[13] Drewing, K., Ramisch, A. & Bayer, F. Haptic, visual and visuo-haptic softness judgments for objects with deformable surfaces. In *World Haptics 2009-Third Joint EuroHaptics Conference and Symposium on Haptic Interfaces for Virtual Environment and Teleoperator Systems* 640–645 (IEEE, 2009).

[14] Han, D. & Keyser, J. Effect of appearance on perception of deformation. In *Proceedings of the 14th ACM SIGGRAPH/Eurographics Symposium on Computer Animation* 37–44 (2015).

[15] Han, D. & Keyser, J. Effect of low-level visual details in perception of deformation. In *Computer Graphics Forum*, vol. 35, 375–383 (Wiley, 2016).

[16] Fakhoury, E., Culmer, P. R. & Henson, B. The effect of indentation force and displacement on visual perception of compliance. In *2015 IEEE World Haptics Conference (WHC)* 88–93 (IEEE, 2015).

[17] Paulun VC, Schmidt F, van Assen JJR, Fleming RW (2017). Shape, motion, and optical cues to stiffness of elastic objects. J. Vis..

[18] Kawabe, T. & Nishida, S. Seeing jelly: Judging elasticity of a transparent object. In *Proceedings of the ACM Symposium on Applied Perception* 121–128 (2016).

[19] Bi, W. & Xiao, B. Perceptual constancy of mechanical properties of cloth under variation of external forces. In *Proceedings of the ACM Symposium on Applied Perception* 19–23 (2016).

[20] Norman JF, Wiesemann EY, Norman HF, Taylor MJ, Craft WD (2007). The visual discrimination of bending. Perception.

[21] Kawabe T, Maruya K, Fleming RW, Nishida S (2015). Seeing liquids from visual motion. Vis. Res..

[22] Piovarči M (2016). An interaction-aware, perceptual model for non-linear elastic objects. ACM Trans. Graph. (TOG).

[23] Bi W, Jin P, Nienborg H, Xiao B (2018). Estimating mechanical properties of cloth from videos using dense motion trajectories: Human psychophysics and machine learning. J. Vis..

[24] Bi W, Jin P, Nienborg H, Xiao B (2019). Manipulating patterns of dynamic deformation elicits the impression of cloth with varying stiffness. J. Vis..

[25] Lucas, B. D. *et al.**An Iterative Image Registration Technique with an Application to Stereo Vision*. IJCAI (1981).

[26] Schmidt F, Paulun VC, van Assen JJR, Fleming RW (2017). Inferring the stiffness of unfamiliar objects from optical, shape, and motion cues. J. Vis..

[27] Burr DC, Ross J, Morrone MC (1986). Seeing objects in motion. Proc. R. Soc. Lond. Ser. B Biol. Sci..

[28] Watamaniuk SN, McKee SP, Grzywacz NM (1995). Detecting a trajectory embedded in random-direction motion noise. Vis. Res..

[29] Salmela VR, Mäkelä T, Saarinen J (2010). Human working memory for shapes of radial frequency patterns. Vis. Res..

[30] Jain A, Zaidi Q (2011). Discerning nonrigid 3D shapes from motion cues. Proc. Natl. Acad. Sci..

[31] Kane D, Bex P, Dakin S (2011). Quantifying, “the aperture problem” for judgments of motion direction in natural scenes. J. Vis..

[32] Van Doorn A, Koenderink J (1982). Spatial properties of the visual detectability of moving spatial white noise. Exp. Brain Res..

[33] Nakayama K, Silverman GH, MacLeod DI, Mulligan J (1985). Sensitivity to shearing and compressive motion in random dots. Perception.

[34] Fleming RW (2014). Visual perception of materials and their properties. Vis. Res..

[35] Fleming RW (2017). Material perception. Annu. Rev. Vis. Sci..

[36] Nishida S (2019). Image statistics for material perception. Curr. Opin. Behav. Sci..

[37] Kobayashi M, Motoyoshi I (2019). Perceiving natural speed in natural movies. i-Perception.

[38] Kawabe T (2019). Visual assessment of causality in the Poisson effect. Sci. Rep..

[39] Mckee SP, Nakayama K (1984). The detection of motion in the peripheral visual field. Vis. Res..

[40] Bruyn BD, Orban GA (1988). Human velocity and direction discrimination measured with random dot patterns. Vis. Res..

[41] Nover H (2005). A logarithmic, scale-invariant representation of speed in macaque middle temporal area accounts for speed discrimination performance. J. Neurosci..

[42] Marr D (1976). Early processing of visual information. Philos. Trans. R. Soc. Lond. B Biol. Sci..

[43] De Leeuw JR (2015). jsPsych: A javascript library for creating behavioral experiments in a web browser. Behav. Res. Methods.

[44] Bridges D, Pitiot A, MacAskill MR, Peirce JW (2020). The timing mega-study: Comparing a range of experiment generators, both lab-based and online. PeerJ.

