# Supplementary material for: Perceptual judgments for the softness of materials under indentation
Source: Sci Rep. 2022 Feb 2;12:1761. doi: 10.1038/s41598-022-05864-x (PMC8810927; doi:10.1038/s41598-022-05864-x)
Supplement: Supplementary file 2 — Supplementary Information 2. [file 41598_2022_5864_MOESM2_ESM.pdf]

Supplementary Information for

"Perceptual judgments for the softness of materials under indentation"

**Yusuke Ujitoko[1], Takahiro Kawabe[1]**

**[1]NTT Communication Science Laboratories**

## Supplementary Videos: Stimuli presented to observers

The stimuli are video clips that shows the scenes of an elastic material pushed from the top surface by an indentor. The video resolution is 288 x 288 pixels at 29.97 frames per second. In total, there are 75 conditions (3 material compliance levels x 5 depth levels x 5 speed levels)

## Supplementary Figure 1: 14 points used for calculation of optical flow

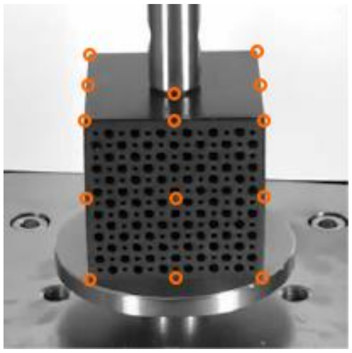

**Supplementary Table 1: Diameter of the holes of the materials**

| | Low compliance | Medium compliance | High compliance |
|---|---|---|---|
| Diameter of first size of hole [mm] | 0.6 | 1.2 | 0.4 |
| Diameter of second size of hole [mm] | 0.5 | 0.65 | 1.8 |

Each column shows the diameter of holes of each material.

**Supplementary Table 2: Spearman's rank correlation coefficient between local motion speed/overall deformation magnitude and idiosyncratic motion features**

| | Local motion speed | Overall deformation magnitude |
|---|---|---|
| Absolute divergence.Mean | **0.954 (p<0.001)** | -0.225 (p=0.052) |
| Absolute divergence.SD | **0.947 (p<0.001)** | -0.200 (p=0.084) |
| Gradient.Mean | **0.940 (p<0.001)** | -0.252 (p=0.029) |
| Gradient.SD | **0.940 (p<0.001)** | -0.205 (p=0.077) |
| Discrete laplacian.Mean | **0.960 (p<0.001)** | -0.212 (p=0.068) |
| Discrete laplacian.SD | **0.948 (p<0.001)** | -0.201 (p=0.084) |
